# Overexpression of CDC2/CyclinB1 in gliomas, and CDC2 depletion inhibits proliferation of human glioma cells *in vitro *and *in vivo*

**DOI:** 10.1186/1471-2407-8-29

**Published:** 2008-01-29

**Authors:** Hua Chen, Qiang Huang, Jun Dong, De-Zhong Zhai, Ai-Dong Wang, Qing Lan

**Affiliations:** 1 Department of Neurosurgery and Brain Tumor Research Laboratory, 2nd Affiliated Hospital, Soochow University; Laboratory of Aging and Nervous Diseases, Soochow University, Suzhou, China

## Abstract

**Background:**

Gliomas are the most common and aggressive primary brain tumors for which unfortunately no effective treatment modalities exist despite advances in molecular biology as the knowledge base to unravel the extremely complex molecular mechanisms of tumorigenesis is limited. In this study an attempt has been made to understand the molecular pathological basis of tumorigenesis which led to an identification of an oncogene, CDC2, and an epigenetic strategy has been evaluated to control the tumorigensis by downregulating this oncogene.

**Methods:**

Tissue microarrays were utilized to investigate the expression of genes in a large number of tumor samples and to identify overexpressed genes which could be potentially causing tumorigenesis. Retroviral vectors expressing short hairpin RNAs (shRNAs) targeted against CDC2 were designed and transducted into human glioma cell line ex vivo in order to downregulate the expression of CDC2. Real-Time PCR was used to determine the level of CDC2 mRNA. Western Blotting was used to determine the level of expression of CDC2 protein as measure to quantify down regulation of CDC2 expression along with use of flow cytometry to investigate effect of shRNAs on cell cycles and detection of apoptosis. Following ex vivo study, viral particles containing small interfering RNA for CDC2 were subsequently injected into xenogeneic graft tumor of nude mice and the weight of human glioma xenografts, survival and resulting phenotypic changes of target gene were investigated.

**Results:**

Human glioma tissue microarrays indicated the positive expression rates of CDC2/CyclinB1 with a positive correlation with pathologic grades (r = 0.982, r = 0.959, respectively). Retroviral vectors expressing short hairpin RNAs (shRNAs) against CDC2 caused efficient deletion of CDC2, cellular G2/M arrest concluding in apoptosis and inhibition of proliferation in human glioma cells U251 and SHG-44 cell lines ex vivo. And the viral particles containing small interfering RNA for CDC2 were subsequently injected into subcutaneous and intracranial xenogeneic graft tuomrs of nude mice. For subcutaneous tumors, injection of CDC2-shRNA retroviruses significantly decreased tumor weight and volume compared with control. Immunohistochemistry indicated that CDC2 are negative and TUNEL are positive in tumors treated with recombinant retrovirus. For mice implanted with intracranial gliomas, treatment of CDC2-shRNA retroviruses increased survival times compared with control.

**Conclusion:**

CDC2 gene plays an important role in the proliferation of human gliomas. Downregulation of CDC2 could potentialy inhibit human gliomas cells growth ex vivo and in vivo. From these results, it was suggested that CDC2 might be a potential target on gene therapy of human gliomas.

## Background

Abnormalities in cell cycle regulation are reported to be strongly associated with tumorigenesis and progression of tumors. The cell cycle is a complex process with myriad genes involved and elaborate and complex signaling mechanisms to allow for this critical cellular process. In eukaryotes, the entry into mitosis is regulated by the activation of CDC2/Cyclin B1 complex (M-phase promoting factor, MPF). CDC2 is known as an active sub-unit of the MPF. It is inhibited by Cyclin-dependent kinase inhibitors (CKI). Appropriate regulation of CDC2 is essential for the entry into mitosis. Increasingly numerous results have demonstrated overexpression of CDC2/Cyclin B1 in various tumors however there is still no report of CDC2/Cyclin B1 expression in clinical samples from patients with gliomas.

In the present study, we investigated the relationship of gene expression profiles with malignant progression of human glioma. Tissue microarrays (TMAs) including I–IV grade clinical glioma samples was used to determine the prognostic effect of the CDC2/Cyclin B1 expression in gliomas on different grades. To reveal the roles of CDC2/Cyclin B1 in human malignant gliomas, CDC2 in glioma cell line, it was down-regulated by retrovirus vectors expressing short hairpin RNAs (shRNAs) ex vivo and in vivo.

## Methods

### Clinical information

**Figure 1:**
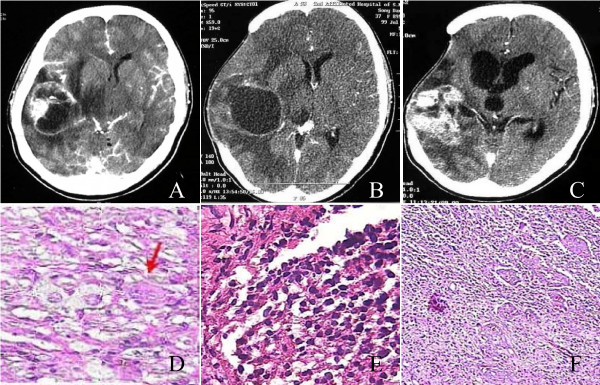
Clinical information of 37-year-old female patient. A, Brain enhanced CT scan demonstrated an irregularly enhanced tumor node with cyst in right temporal lobe. B showed 1st recurrence tumor relapsed and invaded posteriorly, superiorly to parietal lobe. C showed 2nd recurrence tumor was solid lesion with obvious enhancement. D, primary tumor (HE staining, magnification 400×): neoplastic neurons, neoplastic astrocytes and binucleate neurons (↑) can be seen. Pathological diagnosis was ganglioglioma (WHO grade II). E, 1st recurrent tumor (HE staining, magnification 400×): Neop1astic neurons disappeared, while neoplastic glial cells increased with high density. Nuclei heteromorphism can be seen and chromatin increased obviously. Pathological diagnosis was anaplastic astrocytoma (WHO grade III). F, 2nd recurrent tumor (HE staining, magnification 100×): Microscopic field was crowded with neoplastic astrocytes. And pseudopalisade formation and multinucleated giant cells were found. Pathological diagnosis was glioblastoma multiform (WHO grade IV).

Tumor specimens were obtained with informed consent from a 37-year-old female patient who underwent initial surgical removal in January 1999 and subsequent recurrent resections of a right temporal tumor July 1999 and February 2001 at the 2nd Affiliated Hospital of Soochow University (Suzhou, China) and did not receive chemotherapy and radiation between these stages of progression. The tumor was diagnosed as ganglioglioma (WHO grade I), anaplastic astrocytoma (WHO grade III) and glioblastoma (WHO grade IV) on paraffin embedded sections (Figure 1). Normal brain tissues were obtained from the same patient when she was undergoing partial removal of the right occipital lobe for cerebral decompression. Part of the fresh tissue was used for initiation of tissue culture and the remaining part was snap frozen in liquid nitrogen and stored at -80°C for subsequent molecular cytogenetic analysis.

### Cell Culture

Human glioma cell line SHG-44 (P53 mut) was derived from a poorly differentiated astrocytoma of the left frontal lobe of a 32-year-old woman who was undergoing surgical removal of a right temporal tumor at the Department of Neurosurgery, the 2nd Affiliated Hospital of Soochow University School of Medical (Suzhou, China). It was the first established human glioma cell line in China according to the library and information system of the Chinese Academy of Sciences, and has been widely used in human glioma research in China [1-3]. Human glioma cell line U251 (P53 mut) was obtained from the Institute of Biochemistry and Cell Biology, Shanghai Institutes for Biological Sciences, Chinese Academy of Sciences (Shanghai, China). Phoenix cells were the gift from Dr. Gu JM (Vanderbilt University, Nashville, Tennessee). All cell lines were cultured in Dulbecco's Modified Eagle Medium (Invitrogen, California, U.S.) supplemented by 10% heat-inactivated fetal calf serum (Si Ji Qing, Hangzhou, China).

### Culture of Brain tumor stem cell spheres, neural stem cell spheres and multicellular tumor spheres

Human glioma tissues were obtained from a 52-year-old female patient who underwent two operations within 2 months for rapid relapse of human malignant gliomas. Both primary and recurrent lesions were pathologically diagnosed as mixed glioma consisting of anaplastic ependymoma and astrocytoma. Human fetal brain tissue was obtained from consent-informed women who received induced abortion in accordance with the protocol approved by Ethical guidelines for the use of human embryonic or fetal tissue for experimental and clinical neurotransplantation and research of Network of European CNS Transplantation and Restoration (NECTAR) [4].

The fresh tumor specimens was washed, deprived of vessels, acutely dissociated in oxygenated artificial cerebrospinal fluid and subjected to enzymatic dissociation as described previously [5]. Tumor cells were then re-suspended in serum-free DMEM/F-12 containing human recombinant N2, EGF and bFGF (20 ng/ml; Invitrogen, California, U.S.), and plated at a density of 3 × 106 live cells/60-mm plate. Cells were fed by changing half of the medium every 3 days. Brain tissue from a 1-month-old abortive dead embryo was subjected to the same procedure as described above to get neural stem cell spheres [6]. SHG44 and U251 cells were planted in liquid media over an agar base to form multicellular tumor spheres [7].

### Construction of tissue microarray and Immunohistochemical analysis

A total of 71 gliomas of the cerebral hemisphere, resected at the 1st and 2nd affiliated Hospital of Soochow University were studied. These tumors were from 40 men and 31 women, ranging in age from 4 to 80 (mean, 41.04) years at diagnosis. All of these patients did not receive chemotherapy and radiation before surgical removal. Tumors were classified and graded using the World Health Organization (WHO) scheme (2000) (grades I–IV) [8]. Eleven normal brain tissues were gathered from consent-informed individuals who underwent partial removal of the temporal lobe for cerebral decompression. SHG44 multicellular tumor spheres (n = 4), GBM multicellular tumor spheres (n = 4), brain tumor stem cell spheres (n = 4) and neural stem cell spheres (n = 4) were centrifuged and embedded in melted 3% agarose gel to form agarose cell blocks (ACBs) [9]. Tissue microarray (TMA) technique was used for immunohistochemical study [10]. The tumor samples and cell blocks were fixed in 4% phosphate buffered formaldehyde and processed into paraffin blocks with standard methods. Histologically representative tumor regions of hematoxylin-eosin (HE) stained slides were selected by a neuropathologist (Professor Liu zhen yan) and corresponding areas were sampled in tissue microarray blocks with the aid of manual tissue microarrayer (Bo Nan Biotechnology Inc. Shanghai, China) according to the manufacturer's instruction. The sample diameter of the tissue core in the microarray block was 600 μm.

Tissue microarray sections were washed in PBS and incubated for 1 h with rabbit anti CDC2 polyclonal antibody (Santa Cruz Biotechnology), rabbit anti CyclinB1 polyclonal antibody (Santa Cruz Biotechnology) or with 1% BSA-PBS as a negative control. After washing, the slides were incubated with biotinylated anti-rabitt immunoglobulin (LSAB) for 30 min, washed again, and incubated with horseradish peroxidase-conjugated streptavidin for 30 min. The reaction was revealed by 3, 3'-diaminobenzidine and counterstained with hematoxylin.

### Evaluation of slides

Antigen expression was defined as the presence of nuclear staining on tumor cells for CDC2, CyclinB1 and TUNEL. An immunoreactive score (IRS) was applied. The IRS (negative (-), 0~2; weak positive (+), 3~4; positive (++), 5~6; strong positive (+++), >7) was the product of staining intensity (graded between 0 and 3) and the percentage of positive cells (graded between 0 and 4: 0, ≤5%; 1, 6%~25%; 2, 26%~50%; 3, 51%~75%; 4, ≥75%) [11]. Five vital tumor fields were evaluated (magnifications 400×) and a final mean score for each tumor was achieved.

### Construction of recombinant retroviral vectors expressing short hairpin RNAs and transfection *in vitro*

**Figure 2:**
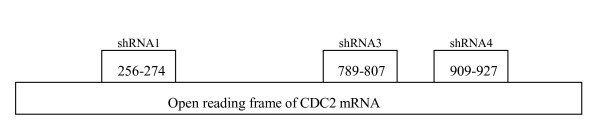
Schematic depiction of three shRNA: Three pairs of oligonucleotides were designed and were synthesized by BIO BASIC Inc (Shang Hai, China). shRNA1 corresponds to positions 256–274 of the CDC2 open reading frame, shRNA3 to positions 789–807 and shRNA4 to positions 909–927. the following were the sequence of three pairs of oligonucleotides: Oligo 1:forward 5'-GAT CCC CGG GGT TCC TAG TAC TGC AAT TCA AGA GAT TGC AGT ACT AGG AAC CCC TTT TTA-3'; reverse5'-AGC TTA AAA AGG GGT TCC TAG TAC TGC AAT CTC TTG AAT TGC AGT ACT AGG AAC CCC GGG-3'; Oligo 3:forward 5'-GAT CCC CGG GCA CTC CCA ATA ATG AAT TCA AGA GAT TCA TTA TTG GGA GTG CCC TTT TTA-3', reverse 5'-AGC TTA AAA AGG GCA CTC CCA ATA ATG AAT CTC TTG AAT TCA TTA TTG GGA GTG CCC GGG-3'; Oligo 4: forward 5'-GAT CCC CCT TGG ATT TGC TCT CGA AAT TCA AGA GAT TTC GAG AGC AAA TCC AAG TTT TTA-3', reverse5'-AGC TTA AAA ACT TGG ATT TGC TCT CGA AAT CTC TTG AAT TTC GAG AGC AAA TCC AAG GGG-3'.

The pSUPER.retro.puro purchased from OligoEngine was a vector system for expression of short interfering RNA. Recombinant retroviral vectors expressing short hairpin RNAs were constructed according to the product manual. Three pairs of oligonucleotides were designed. The shRNA1, 3 corresponds to positions 256–274, 789–807 of the CDC2 open reading frame, respectively. The oligoengine workstation software gives the effect target corresponds to positions 908 of the CDC2 open reading frame. But we designed C4 target corresponds to positions 909 (909–927). We want to know whether it was an "inactive target" (Figure 2). These forward and reverse oligos were annealed and cloned into the vector between the unique BglII and HindIII enzyme sites. The presence of the correct insert of recombinant pSUPER.retro vectors (C1, C3, C4) were confirmed by sequencing (BIO BASIC Inc, Shanghai, China; Sequencing primer 5'-GGA AGC CTT GGC TTT TG-3').

Glioma cell lines were transfected with recombinant retroviral vectors using a modified Lipofectamine™ 2000 protocol (Invitrogen, California, U.S.), in which the quantity of plasmid DNA was double of the recommended quantity in order to achieve the highest transfection efficiency. Cells were harvested 48 h after recombinant retroviral vectors transfection for RT-PCR, Western blots, cell cycle analysis and apoptosis analysis.

### Cell proliferation assay *in vitro*

SHG44 and U251 cells were plated in 24-well plates (Corning, NY, U.S.A) at 1.5 × 105 per well. Twenty four hours after transfection, cells were transferred into a 50 ml cell culture flask (Corning). Time kinetics of cell proliferation was conducted at 24, 48, 72 and 96 h after transfection with recombinant retroviral vectors. Cells were harvested with 0.25% trypsin, 1 mM EDTA in phosphate buffered saline (PBS) and stained with Trypan blue. The number of cells was counted with a microscope using a haemacytometer.

### RT-PCR and real time PCR

Total RNA was isolated from cells using TRIzol reagent (Gibco, California, U.S.A) according to the manufacturer's instructions. Total RNA (5 μg) was reverse transcribed using 1 μg oligodT primer with MMLV reverse-transcriptase (Promega) in a 40 μ1 reaction volume containing 1.25 mM deoxyribonucleoside triphosphate (dNTP) at 42°C. PCR was performed using 1 μl of cDNA in 50 μl PCR reaction buffer containing 15 pmol of each primer, 0.1 mM dNTP, and 0.3 units Taq polymerase (Promega). Primer sequences used and conditions of these reactions were as follows: CDC2: sense 5'-GGT TCC TAG TAC TGC AAT TCG-3'; antisense 5'-TTT GCC AGA AAT TCG TTT GG-3', (94°C 1 min, 51°C 1 min, 72°C 1 min, ×30 cycles, 709 bp as PCR product), Cyclinb1: sense5'-CAG TCA GAC CAA AAT ACC TAC TGG GT-3'; antisense 5'-ACA CCA ACC AGC TGC AGC ATC TTC TT-3', (94°C 30 s, 54°C 30 s, 72°C 30 s ×30 cycles, 191 bp as PCR product), β-actin (internal control): sense 5'-TCC TGT GGC ATC CAC GAA ACT-3'; antisense 5'-GAA GCA TTT GCG GTG GAC GAT-3'(314 bp as PCR product). The products were electrophoresed through 2% agarose gel.

cDNA was used to perform real-time quantitative PCR using MJ Research PTC-100 with TaKaRa Ex Taq R-PCR Version 2.1 (TaKaRa, Da lian, China) and EvagreenTM (Biotium, Inc., CA, U.S.A) according to product's instruction. Primer sequences are shown as follows: CDC2: sense 5'-CAG TCT TCA GGA TGT GCT TAT GC-3'; antisense 5'-GAG GTT TTA AGT CTCTGT GAA GAA CTC-3'. β-actin: sense 5'-AGC GAG CAT CCC CCA AAG TT-3'; antisense 5'-GGG CAC GAA GGC TCA TCA TT-3'.

### Protein extraction and western blot analysis

Cells were harvested using trypsin:EDTA and lysed in TRIzol reagent (Gibco). Proteins were precipitated from the phenol-ethanol supernatant with isopropyl alcohol obtained after precipitation of DNA with ethanol and dissolved in 1% SDS by pipetting. Protein concentrations in solutions were determined using the Bradford protein assay. PageRuler™ Prestained Protein Ladder (5 μl) (Fermentas Inc. MD, U.S.A) and protein solutions containing 40 μg per lane were electrophoresed using SDS-PAGE (15% polyacrylamide gel) and blotted onto nitrocellulose (NC) membrane. Membranes were blocked with 5% fat-free milk solution. Samples were probed with 1:200 dilution of a Rabbit polyclonal antibodies against CDC2 (Santa-Cruz Biotechnology, Inc., Santa Cruz) and a 1:5000 dilution of goat anti-rabbit HRP-conjugated IgG (Hua mei Chemical Corp., China). Rabbit monoclonal antibody against GAPDH (Affinity BioReagents, Inc.) was used as a loading control. Bands were revealed using ECL kit (Pu fei Chemical Corp., Shang hai, China).

### Analysis of cell cycle and apoptosis

Cell viability was assessed by Trypan blue staining. Cell cycle was analysed using a FACScan flow cytometer (Becton Dickinson). Briefly, cells were washed with PBS, treated with RNase A, and stained with PI (Sigma). Apoptosis was assessed using rh Annexin V FITC Kit according to manufacturer's instructions (Bender Medsystems). The data were acquired using Cell Quest V.3.3 software (BD). To detect apoptotic cells in xenogeneic graft tumors, sections were analyzed by terminal deoxynucleotidyl transferase-mediated deoxyuridine triphosphate-biotin nick end-labeling (TUNEL) (Roche Molecular Biochemicals), according to the manufacturer's instructions.

### Viral packaging

The effective recombinant retroviral vectors were packaged in Pheonix cells by standard calcium phosphate transfection protocol [12]. Virus supernatant was collected 48 hours post-transfection, filtered to remove nonadherent cells and cellular debris, frozen in small aliquots on dry ice, and stored at -80 C. Detection of virus titer was refer to the methods of Short Protocols in Molecular Biology [13].

### Mouse Models of Gliomas and Recombinant Retrovirus Injections

Nude mice with nu/nu gene from the NC strain (Fujita Health University, Japan) were bred and maintained in our Specific Pathogen Free Animal Care Facility. Female mice (6- to 8-week-old) were used in this study. Subcutaneous injections of 5 × 106 tumor cells in 0.2 ml solution were made into the flanks of recipient nude mice, as described previously [14]. Eight mice were included in control and experimental groups. When the diameter of xenogeneic graft tumors was about ~0.5 cm, recombinant retrovirus was directly injected into xenogeneic graft tumors by using the following parameters: 10 μl per site, 10 μl infusion rate/min, 100 μl per one injection, injection per 3 days and total 3 times (total 300 μl). Twenty days after the last injection, the difference of tumor weights between control group and experimental group was compared.

To determine if CDC2-depletion prolonged animal survival, 1 mm3 SHG44 xenogeneic graft tumor tissue was injected into the frontal lobe of 6-week-old nude mice by using the following parameters: 0.5-mm anterior, 2.5-mm lateral, 2.5-mm depth. After 3 days, animals were treated with a single intratumoral injection of PBS and recombinant C3 retrovirus (n = 8/group 20 μl per mice, 0.5 μl/min infusion rate).

### Statistical methods

The relationship between expression rates of CDC2 and pathologic grades of human gliomas was analyzed with correlation coefficient. The Spearman's Rank Correlation was used to measure the correlation between CDC2 and CyclinB1 expression in gliomas. Statistical analyses ex vivo were performed using the Student's t test. Statistical analyses in vivo were performed using the Wilcoxon signed-rank test. All the reported P-values are 2-sided and a value of P < 0.05 was considered statistically significant.

### Statement of ethics

Research that is reported in the manuscript has been performed with the approval of ethics committee of 2nd affiliated hospital of Soochow University. Animal studies were approved by the Soochow University of Animal Care and Use Committee and followed internationally recognized guidelines.

## Results

### Expression of CDC2 in one recurrence brain tumor case

**Figure 3:**
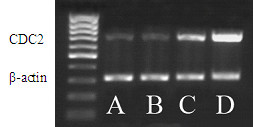
Expression of CDC2 in one recurrence brain tumor case was detected by RT-PCR. CDC2 gradually increased with malignant grade, β-actin served as an equal loading control. Normal brain tissue (lane A), primer ganglioglioma (lane B), first recurrence anaplastic astrocytoma (lane C), second recurrence glioblastoma multiform (lane D)

Expression of CDC2 in primary ganglioglioma (WHO grade II) was as low as that in normal brain tissue, and increased by 2.5 fold and 5.3 fold in the first and second recurrence tumor respectively (Figure 3).

### Expression of CDC2 and CyclinB1 in different grade brain tumors

**Figure 4:**
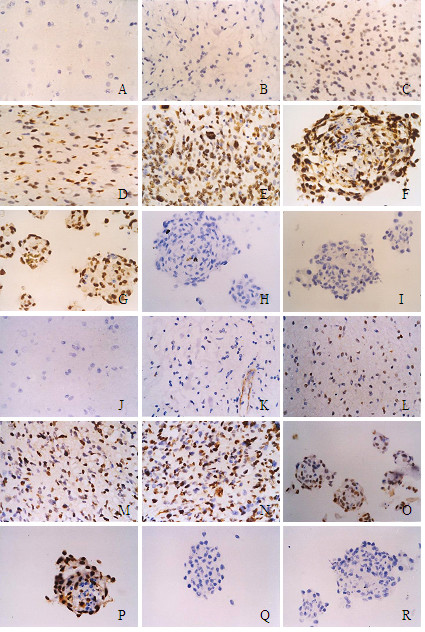
Immunoreactive score of CDC2/CyclinB1 in different grade brain tumor and normal brain tissues. A-I: CDC2 immunohistochemical images, J-R: CyclinB1 immunohistochemical images. CDC2/CyclinB1 was expressed in the cytoplasm of tumour cells. A/J: Normal adult brain tumor (-); B/K: Polycystic astrocytoma (-); C/L: Fibrillary astrocytoma (+); D/M: Anaplastic Astrocytoma (++); E/N: medulloblastoma (+++); F/O: SHG44 multicellular tumor spheres (+++); G/P: GBM multicellular tumor spheres (+++); H/Q: Neural stem cell spheres (-); I/R: Brain tumor stem cells spheres (-).

In CDC2/CyclinB1 immunostaining, CDC2/CyclinB1 was expressed in the nuclei of tumor cells. Histologically, high-grade gliomas tended to express high CDC2/CyclinB1 protein levels than that in lower grade gliomas. The immunoreactivity of CDC2/CyclinB1 showed appreciably strong staining intensity among the high-grade brain tumors. Statistically significant difference in CDC2 expression was observed between grade I and III, grade I and IV, grade II and III, grade II and IV (P = 0.005, P = 0.008, P = 0.009, P = 0.020, respectively). Also, statistically significant difference in CyclinB1 expression was observed among different grade. Furthermore, the positive expression rates of CDC2/CyclinB1 were positively correlated with the pathologic grades (r = 0.982, r = 0.959, respectively) (Figure 4, Table 1). Expression of CDC2/CyclinB1 was very high in SHG44 and GBM, but was negative in brain tumor stem cell spheres and neural stem cell spheres. The positive expression rates of CDC2 correlated with CyclinB1 (r = 0.900, P < 0.01).

**Table 1:** Expression rate of CDC2/CyclinB1 in 98 specimens

Specimens	Case	CDC2 expression (case)				Percentage of Positive cells (%)
			
		Negative (-)	Weak Positive (+)	Positive (++)	Strong Positive (+++)	
Normal adult brain tumor	11	9	2	0	0	18.2
Grade I	9	7	2	0	0	22.2
Polycystic astrocytoma	7	6	1	0	0	
Choroid plexus papilloma	2	1	1	0	0	
Grade II	25	15	8	2	0	40.0
Fibrillary astrocytoma	16	11	4	1	0	
Protoplasmic astrocytoma	3	2	1	0	0	
Gemistocytic astrocytoma	1	0	1	0	0	
Oligodendroglioma	4	1	2	1	0	
Ependymoma	1	1	0	0	0	
Grade III	23	7	8	5	3	69.6
Anaplastic Astrocytoma	18	6	7	3	2	
Anaplastic oligodendroglioma	2	0	1	0	1	
Anaplastic ependymoma	3	1	0	2	0	
Grade IV	14	3	3	4	4	78.6
Glioblastoma	6	0	2	2	2	
Glioma sarcomatosum	1	0	0	1	0	
Medulloblastoma	6	2	1	1	2	
Primitive neuroectodermal tumor	1	1	0	0	0	
Multicellular tumor spheres	8	1	0	3	4	87.5
Brain tumor stem cell spheres	4	4	0	0	0	0.0
Neural stem cell spheres	4	4	0	0	0	0.0

### Downregulation of CDC2 with recombinant C1 or C3 inhibits the proliferation in SHG44 and U251 cells

**Figure 5:**
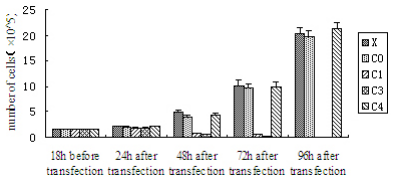
Graph showing cell growth treated with recombinant C1, C3, C4, blank retroviral vector (C0) and transfection reagent oligofectamine alone (X).

As CDC2 is one of the key components for the regulation of cell proliferation, the anti-proliferative effect of recombinant C1 or C3 on human glioma cell lines was analysed. Compared to control treatment with blank retroviral vector (C0) and transfection reagent oligofectamine alone (X), transfection with recombinant C1 or C3 reduced the number of SHG44 cells by 82%, 92% at 48 h, and 95%, 97% at 72 h, respectively. At 96 h, no SHG44 cells were detectable with recombinant C1 or C3 treatment. However, treatment with the transfection recombinant C4 had no effect on inhibition of proliferation in SHG44 cells. The result indicated C4 was an "inactive target" (Figure 5). Anti-proliferation of recombinant C1 or C3 was more robust in U251 cells. The number of U251 cells was reduced by 90% (C1) and 93% (C3) at 24 h, and 94%, 98% at 48 h, respectively. At 72 h, no U251 cells were detectable with recombinant C1 or C3 treatment.

### Recombinant C1 or C3 reduce mRNA and protein expression of CDC2 in SHG-44

**Figure 6:**
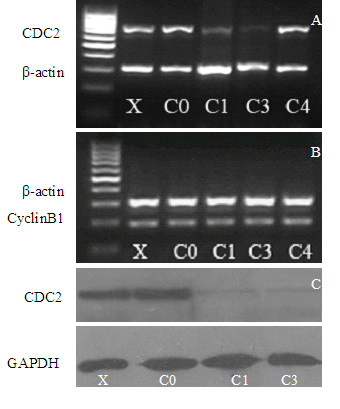
Reduction of CDC2 mRNA and protein in SHG44 cell lines by recombinant C1 or C3. A, B, RT-PCR analysis: SHG44 cells were treated with recombinant C1 or C3, C4, blank retroviral vector (C0) and transfection reagent oligofectamine alone (X) for 48 h, β-actin served as an equal loading control. C, Western blot analysis, the membrane was stained with antibodies against CDC2 and GAPDH. The latter served as an equal loading control.

Due to the tremendous anti-proliferation, we failed to harvest enough cells to perform RT-PCR and Western blot analysis on U251 cells, and therefore analyses were performed on SHG44 cells 48 h after transfection with the recombinant retroviral vectors to examine the effects of the three recombinant retroviral vectors on CDC2 mRNA and protein expression. mRNA level of CDC2 was significantly downregulated after the transfection with recombinant C1 or C3 as compared to negative controls, off-target sequence C4, the blank retroviral vector C0, and transfection reagent alone X (Figure 6A, lanes 1, 4 and 5). None of the recombinant retroviral vectors affected mRNA level of Cyclin B1 (Figure 6B), suggesting that these recombinant C1 C3, and C4 were highly specific. The real-time PCR revealed that mRNA level of CDC2 decreased by 98.3% and 98.5% after transfection with recombinant C1 and C3, respectively (data not shown).

The protein level of CDC2 standardized to the level of GAPDH efficiently decreased by 85% and 88% after transfection with recombinant C1 or C3 (Figure 6C, lanes 3 and 4). Treatment with transfection reagent oligofectamine alone(X) or with control blank recombinant retroviral vector (C0) had almost no effect (Figure 6C, lanes 1 and 2).

### Downregulation of CDC2 induces G2/M arrest in SHG-44 cells

**Figure 7:**
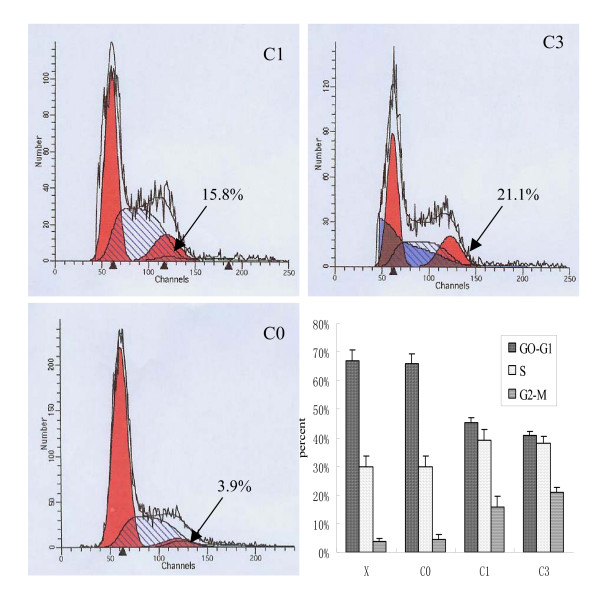
Downregulation of cdc2 induces G2/M arrest in SHG-44 cells. SHG44 cells treated with recombinant C1 or C3 were arrested in G2/M phase. There was no G2/M arrest in cells treated with blank retroviral vector (C0) and transfection reagent oligofectamine alone (X). (n = 3)

As CDC2 is essential for G2/M transition, cell cycle distribution of recombinant C1- or C3-treated cells was analyzed using a FACScan flow cytometer. After incubation for 48 h with 8 μg of recombinant C1 or C3, 15.8% and 21.1% of SHG44 cells were arrested in G2/M phase, respectively, versus 3.9% of control SHG44 cells located in G2/M phase. There was no G2/M arrest in cells treated with lipofectamine (X) alone or cells treated with blank retroviral vector (C0) (Figure 7).

### Treatment with recombinant C1 or C3 triggers apoptosis in SHG-44 cells

**Figure 8:**
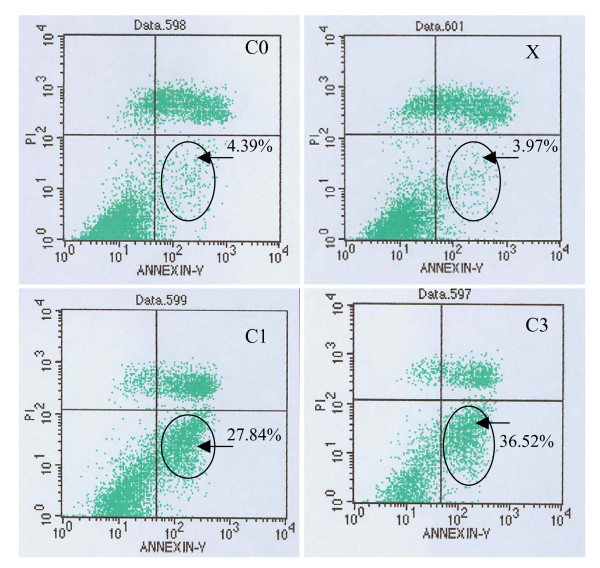
Downregulation of cdc2 induces apoptosis in SHG-44 cells. Graph shows the cell apoptosis treated with recombinant C1 or C3, C0 and X. (n = 3)

In order to examine whether recombinant C1- or C3-treated cells further led to apoptosis, the treated SHG44 cells were stained with annexin V and propidium iodide (PI) and analyzed by flow cytometry. After incubation with recombinant C1 or C3 for 48 h, 27.84% (C1) and 36.52% (C3) of SHG-44 cells were annexin V-positive (early stage of apoptosis), versus 3.97% in lipofectamine (X) alone and 4.39% in blank retroviral vector (C0) treated SHG44 cells (Figure 8).

### Therapeutic potential of CDC2 deletion on human gliomas: *in vivo *studies

**Figure 9:**
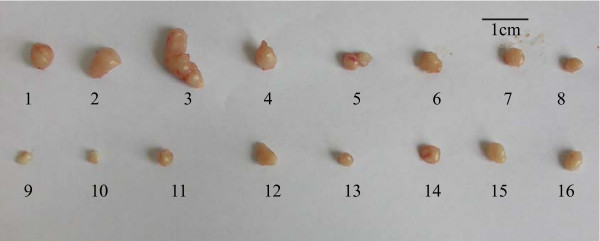
Injection of recombinant retrovirus significantly decreased xenogeneic graft tumor volume. The upper row in the pictures show xenogeneic graft tumor specimens treated with saline as control. The lower row show xenogeneic graft tumor specimens treated with recombinant C3 retrovirus. The lower quartile, median and the upper quartile is 57.6 mg, 79.8 mg, 124.7 mg, respectively in control. The lower quartile, median and the upper quartile is 18.1 mg, 33.5 mg and 42.7 mg respectively in treated (P = 0.003, n = 8).

**Figure 10:**
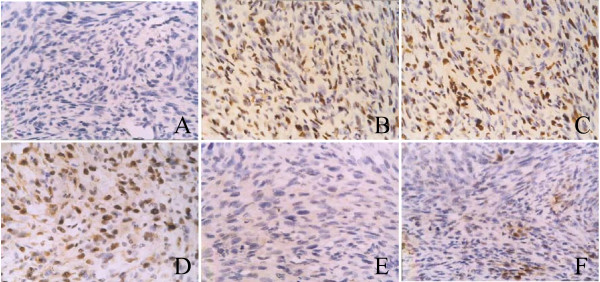
Immunohistochemistry of tissue microarray (magnification 400×). Immunoreactivities of CDC2 (A-C) and TUNEL (D-F) in xenogeneic graft tumor specimens were examined. IRS of CDC2 are negative in tumors treated with recombinant C3 retrovirus (-) (A); positive in tumors treated with saline (++) (B); also positive in NO.12 tumor (C). IRS of TUNEL are positive in tumors treated with recombinant C3 retrovirus (++) (D); negative in tumors treated with saline (-) (E); also negative in NO.12 tumor (-) (F).

**Figure 11:**
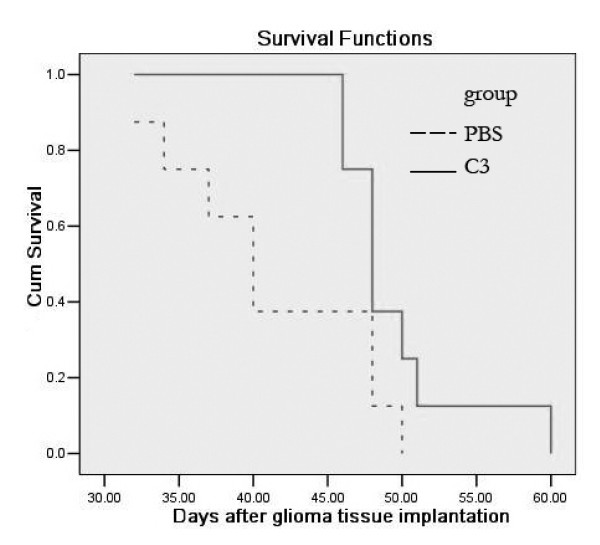
Survival of nude mice after intracranial injection of recombinant C3 retrovirus into established SHG44 gliomas. SHG44 were implanted into the frontal lobe of nude mice. After 3 days, tumors were injected with a single dose of recombinant C3 retrovirus or PBS (n = 8/group). Compared with animals treated with PBS (dash line), a significant increase in survival occurs in those treated with recombinant C3 retrovirus (solid line) (p < 0.05).

Due to the strong inhibitory effect demonstrated ex vivo, recombinant C3 was selected for viral packaging. The impact of recombinant C3 retrovirus on xenogeneic graft tumors was examined in vivo. Virus titer was 2.15 × 107 RCFU (relative colony forming unit). Xenogeneic graft tumors treated with saline as control grew rapidly. In contrast, except tumor NO12, 15, 16, weight and volume of other xenogeneic graft tumors treated with recombinant C3 retrovirus were significantly reduced (Figure 9). Immunohistochemistry indicated that the reduction in the percentage of CDC2 positive cells of the tumors was correlated to the ratio of tumor shrinkage. In contrast, the percentage of TUNEL positive cells was inversely proportional to the ratio of tumor shrinkage (Figure 10). In addition, compared with animals treated with PBS, treatment with recombinant C3 retrovirus resulted in a significant increase (P < 0.05) in animal survival (Figure 11).

## Discussion

Human glioma is the most common and aggressive primary brain cancer with mean patient survival, even with aggressive treatments, including surgery, radiation therapy, and chemotherapy, is typically 12 months or less [8]. Despite progress in research on the molecular aspects of malignant gliomas, the prognosis of these brain tumors continues to be dismal, partly because mechanisms responsible for gliomagenesis and progression remain elusive. In recent years, Ink4a-Arf, KRas, PDGF, PETN, RB, c-Myc and TP53, have been implicated in the pathogenesis and progression of gliomas [15-20], mostly with respect to cell cycle control and signal transduction [21,22]. However, a few studies have reported the molecular mechanism of CDC2/CyclinB1 implicated in gliomagenesis. CDC2 exerts protein kinase activity by constructing a complex with cyclin A, cylin B, and p13suc1 [23,24]. It is known as an active sub-unit of the M-phase promoting factor (MPF) and the M-phase specific histone H1 kinase [25]. Furthermore, CDC2 also plays a role in phosphorylating the retinoblastoma gene product, which regulates the cells cycle by restricting DNA replication [26] at G2/M transition [27]. Appropriate regulation of MPF is essential for the entry into mitosis. Increasing results demonstrate that CDC2/CyclinB1 is involved in checkpoint control, and that its dysregulation could contribute to chromosomal instability in cancer cells through an alteration of spindle checkpoint [28]. p53 gene inhibits cell cycle-dependent expression of CDC2 and Cyclin B1 and consequently inhibits CDC2 kinase [28]. Our data indicated CDC2/CyclinB1 was overexpressed in SHG44 and U251 with p53 mutations. Investigations to date have demonstrated that CDC2/CyclinB1 expression is likely to be correlated with the malignant potential of human tumors [29-31]. Bodey B et al discovered that the expression of CDC2 could become a novel cell proliferation marker in childhood brain tumors [32]. Hence, overexpression of CDC2/CyclinB1 can promote the heterogeneity and malignancy of tumor.

The reason why we chose CDC2/CyclinB1 as a target gene was due to our previous findings that expression level of CDC2 increased with the progression of the malignancy when ganglioglioma transformed into gliobastona multiforme in the same patient [33]. In order to demonstrate the relation in large-scale clinical cases, we constructed human glioma tissue microarrays (TMA). The result of TMA provides the first evidence that expression level of CDC2/CyclinB1 increased with the progression of the malignancy, suggesting that CDC2/CyclinB1 might be of molecular significance in the progression of human gliomas.

We inhibit MPF activation by down-regulation of CDC2 to learn its function in human malignant glioma. Although various inhibitors of CDC2 have been tested as potential candidates for tumor therapy, most of the inhibitors targeting CDC2 lack specificity [34,35]. RNAi is viewed as a powerful tool to define gene function, and a novel therapeutic strategy for treatment of human diseases and conditions including cancer [36]. We demonstrated that inhibition of CDC2 was an effective and specific approach to inhibit the enzymatic activity of MPF, to inhibit proliferation of human malignant glioma cells ex vivo and suppress tumor growth in vivo. Our findings suggest that recombinant retrovirus expressing shRNAs, with its ability to down-regulate CDC2, has potential to serve as a therapeutic agent in the treatment of human malignant gliomas.

Cell cycle analysis indicated that cells transfected with recombinant C1 or C3 were blocked in G2/M phase. This arrest in G2/M phase likely contributes to the observed anti-proliferative and antitumor effects in recombinant C1- or C3-treated tumor cells and xenograft models. In vivo, although the size of most recombinant C3-treated human xenogeneic graft gliomas was reduced markedly, no tumor disappeared as shown ex vivo. Immunohistochemistry also confirmed that inhibition of tumor growth correlated with CDC2 suppression. This indicates that extensive and effective inhibition of CDC2 expression is the key to successful therapy. The approach of directly injecting recombinant retrovirus into the tumor through multi-sites and different time points was unsuccessful because the virus was unable to infect all tumor cells. Improvement of the vector and transport approach is warranted to circumvent the problem, thus gaining success in cancer gene therapy.

It is now widely believed that most tumors including glioblastoma arise from multipotent stem cells, as they have the long lifespan that is required for the accumulation of the many genetic alterations that are found in most cancers [37]. Recent isolation and characterization of brain tumor-initiating cells supports the concept that transformed neural stem cells may seed gliomas [38]. However, the expression of CDC2 was both negative in brain tumor stem cell spheres and neural stem cell spheres. CDC2 was negative in differentiated cells of neural stem cells, versus overexperssion in differentiated cells of brain tumor stem cells (data not show). Thus, overexpression of CDC2 was facilitative but not an initial factor of gliomagenesis. To cure human gliomas, we need to simultaneously target both tumor stem cells and proliferated tumor cells.

## Conclusion

Using sensitive immunohistochemical methods and a large, representative material, we found that the positive expression rates of CDC2/CyclinB1 were positively correlated with the pathologic grades of human gliomas. Overexpression of CDC2 could promote oncogenesis and progression of human gliomas whereas the downregulation of CDC2 expression could inhibit the proliferation, induce G2/M arrest, and trigger apoptosis in human gliomas. Therefore, CDC2 could become a potential target on gene therapy of human gliomas.

## Competing interests

The author(s) declare that they have no competing interests.

## Authors' contributions

HC participated in study design, carried out most of the experiments, and drafted the manuscript.

QH conceived of the study, participated in its design and coordination and participated in manuscript preparation.

JD participated in study, provided statistical assistance and critically revised the manuscript. DZZ carried out the immunoassays and participated in the critical revision of the manuscript.

ADW participated in the molecular genetic studies and the critical revision of the manuscript.

QL participated in the coordination of the study and the critical revision of the manuscript.

All authors read and approved the final manuscript.

## Pre-publication history

The pre-publication history for this paper can be accessed here:



## References

[B1] Du ZW (1984). Establishment of human malignant glioma cell line (SHG-44) and observation on its characteristics. Chinese Journal of Oncology.

[B2] Li XN, Du ZW, Huang Q, Wu JQ (1997). Growth-inhibitory and Differentiation-inducing Activity of Dimethylformamide in Cultured Human Malignant Glioma Cells. Neurosurgery.

[B3] Li XN, Du ZW, Huang Q, Wu JQ (1996). Modulation effects of hexamethylene bisacetamide on growth and differentiation of curtured human malignant glioma cells. J Neurosurg.

[B4] Boer GJ (1994). Ethical guidelines for the use of human embryonic or fetal tissue for experimental and clinical neurotransplantation and research. Network of European CNS Transplantation and Restoration (NECTAR). J Neurol.

[B5] Singh SK, Clarke ID, Terasaki M, Bonn VE, Hawkins C, Squire J, Dirks PB (2003). Identification of a Cancer Stem Cell in Human Brain Tumors. Cancer Res.

[B6] Zhang QB, Ji XY, Huang Q, Dong J, Zhu YD, Lan Q (2006). Differentiation profile of brain tumor stem cells: a comparative study with neural stem cells. Cell Research.

[B7] Yuhas JM, Li AP, Martinez AO, Ladman AJ (1977). A simplified method for production and growth of multicellular tumor spheroids. Cancer Res.

[B8] Kleihues P, Cavenee WK, (eds) (2000). World Health Organization Classification of Tumours-Pathology and Genetics – Tumors of the Nervous System. Lyon.

[B9] Mansy SS (2004). Agarose cell block: innovated technique for the processing of urine cytology for electron microscopy examination. Ultrastructural Pathology.

[B10] Kononen J, Bubendorf L, Kallioniemi A, Bärlund M, Schraml P, Leighton S, Torhorst J, Mihatsch MJ, Sauter G, Kallioniemi OP (1998). Tissue microarrays for high-throughput molecular profiling of tumor specimens. Nat Med.

[B11] Burkhardt M, Mayordomo E, Winzer KJ, Fritzsche F, Gansukh T, Pahl S, Weichert W, Denkert C, Guski H, Dietel M, Kristiansen G (2006). Cytoplasmic overexpression of ALCAM is prognostic of disease progression in breast cancer. J Clin Pathol.

[B12] Sambrook J, Fitsch EF, Maniatis T (1989). Molecular Cloning: A Laboratory Manual.

[B13] Ausubel FM, Brent R, Kingston RE (2002). Short Protocols in Molecular Biology. Short protocols in molecular biology.

[B14] Huang Q, Du ZW, Xu GD, Liu ZY, Guo YH, Chen GL, Ma WX, Tan QY, Xu QN, Li B (1987). Establishment of human glioma cell line – nude mice solid tumor model NHG-1 and its characteristics. Chinese Journal of Oncology.

[B15] Dai C, Celestino JC, Okada Y, Louis DN, Fuller GN, Holland EC (2001). PDGF autocrine stimulation dedifferentiates cultured astrocytes and induces oligodendrogliomas and oligoastrocytomas from neural progenitors and astrocytes in vivo. Genes Dev.

[B16] Ding H, Roncari L, Shannon P, Wu X, Lau N, Karaskova J, Gutmann DH, Squire JA, Nagy A, Guha A (2001). Astrocyte-specific expression of activated p21-ras results in malignant astrocytoma formation in a transgenic mouse model of human gliomas. Cancer Res.

[B17] Holland EC, Celestino J, Dai C, Schaefer L, Sawaya RE, Fuller GN (2000). Combined activation of Ras and Akt in neural progenitors induces glioblastoma formation in mice. Nat Genet.

[B18] Jensen NA, Pedersen KM, Lihme F, Rask L, Nielsen JV, Rasmussen TE, Mitchelmore C (2003). Astroglial c-Myc overexpression predisposes mice to primary malignant gliomas. J Biol Chem.

[B19] Uhrbom L, Dai C, Celestino JC, Rosenblum MK, Fuller GN, Holland EC Ink4a-Arf loss cooperates with KRas activation in astrocytes and neural progenitors to generate glioblastomas of various morphologies depending on activated Akt. Cancer Res.

[B20] Watanabe T, Yokoo H, Yokoo M, Yonekawa Y, Kleihues P, Ohgaki H (2001). Concurrent inactivation of RB1 and TP53 pathways in anaplastic oligodendrogliomas. J Neuropathol Exp Neurol.

[B21] Dai C, Holland EC (2003). Astrocyte differentiation states and gliomas formation. Cancer Journal.

[B22] Hulleman E, Helin K Molecular mechanisms in gliomagenesis. Adv Cancer Res.

[B23] Draetta G, Brizuela L, Potashkin J, Beach D (1987). Identification of p34 and p13, human homologues of the cell regulators of fission yeast encoded by cdc2+ and suc1+. Cell.

[B24] Nigg EA (1995). Cyclin-dependent protein kinases: key regulators of the eukaryotic cell cycle. Bioessays.

[B25] Arion D, Meijer L, Brizuela L, Beach D (1988). Cdc2 is a component of the M phase-specific histone H1 kinase: evidence for identity with MPF. Cell.

[B26] Goodrich DW, Wang NP, Qian YW, Lee EY, Lee WH (1991). The retinoblastoma gene product regulates progression through the G1 phase of the cell cycle. Cell.

[B27] Lees JA, Buchkovich KJ, Marshak DR, Anderson CW, Harlow E (1991). The retinoblastoma protein is phosphorylated on multiple sites by human cdc2. EMBO.

[B28] Park M, Chae HD, Yun J, Jung M, Kim YS, Kim SH, Han MH, Shin DY (2000). Constitutive Activation of Cyclin B1-associated cdc2 Kinase Overrides p53-mediated G2-M Arrest. Cancer Res.

[B29] Fluge O, Bruland O, Akslen LA, Lillehaug JR, Varhaug JE (2006). Gene expression in poorly differentiated papillary thyroid carcinomas. Thyroid.

[B30] Hansel DE, Dhara S, Huang RC (2005). CDC2/CDK1 expression in esophageal adenocarcinoma and precursor lesions serves as a diagnostic and cancer progression marker and potential novel drug target. Am J Surg Pathol.

[B31] Ito Y, Takeda T, Sakon M, Monden M, Tsujimoto M, Matsuura N (2000). Expression and prognostic role of cyclin-dependent kinase 1 (cdc2) in hepatocellular carcinoma. Oncology.

[B32] Bodey B, Siegel SE, Kaiser HE (2002). Expression of proline-directed protein kinase, (p34cdc2/p58cyclin A), a novel cell proliferation marker in childhood brain tumors. In Vivo.

[B33] Huang Q, Dong J, Wang AD, Shao NY, Sun JY, Li XN, Lan Q, Hu GX (2003). Establishment of malignant progression associated gene expression profiles in human brain gliomas. Zhonghua Zhong Liu Za Zhi.

[B34] Davies TG, Bentley J, Arris CE, Boyle FT, Curtin NJ, Endicott JA, Gibson AE, Golding BT, Griffin RJ, Hardcastle IR, Jewsbury P, Johnson LN, Mesguiche V, Newell DR, Noble ME, Tucker JA, Wang L, Whitfield HJ (2002). Structure-based design of a potent purine-based cyclin-dependent kinase inhibitor. Nat Struct Biol.

[B35] Knockaert M, Lenormand P, Gray N, Schultz P, Pouysségur J, Meijer L (2002). p42p44 MAPKs are intracellular targets of the CDK inhibitor purvalanol. Oncogene.

[B36] Silva J, Chang K, Hannon GJ, Rivas FV (2004). RNA-interference-based functional genomics in mammalian cells: reverse genetics coming of age. Oncogene.

[B37] Recht L, Jang T, Savarese T, Litofsky NS (2003). Neural stem cells and neurooncology: Quo vadis?. J Cell Biochem.

[B38] Vescovi AL, Galli R, Reynolds BA (2006). Brain tumour stem cells. Nat Rev Cancer.

